# High-performance one-dimensional thermoelectric materials: polyyne chains and their derivatives†

**DOI:** 10.1039/d4na00998c

**Published:** 2025-05-22

**Authors:** Karthik H. J., Swastibrata Bhattacharyya

**Affiliations:** a Department of Physics, Birla Institute of Technology and Science Pilani Zuarinagar Goa 403726 India swastibratab@goa.bits-pilani.ac.in

## Abstract

Thermoelectricity has emerged as a crucial field in response to pressing environmental concerns and the increasing demand for efficient waste energy conversion. With the miniaturization of devices, there is a demand for smaller material systems with minimal energy dissipation and comparable efficiencies for converting heat into electricity. This study proposes a low-dimensional pure carbon-based system, specifically a one-dimensional (1D) polyyne chain and its derivatives (two, three, and four chains), for thermoelectric applications. We investigated the optimization of the thermoelectric figure of merit (*ZT*) through doping and strain effects at different temperatures. Thermodynamic and structural stability analyzes, including formation energy analyzes, phonon dispersion studies, and *ab initio* molecular dynamics simulations reveal that the material maintains its structural integrity under prolonged high temperature conditions, making it a promising candidate for practical applications. For n-type doping, we achieve a maximum *ZT* of 3.06 for one polyyne chain at 700 K. The corresponding maxima for the two, three and four chains are 1.26, 1.65, and 1.60 respectively. Additionally, we examine cumulative lattice thermal conductivity as a function of phonon frequency and mean free path, along with other phonon properties, such as heat capacity, phonon lifetime, and group velocity, in detail, for different temperatures. The findings underscore the potential of polyyne chain systems in enhancing the efficiency of thermoelectric devices, thus contributing to the advancement of energy harvesting technologies.

## Abstract 2

Polyyne chain derivatives – pathway to enhanced thermoelectric performance.

## Graphical abstract

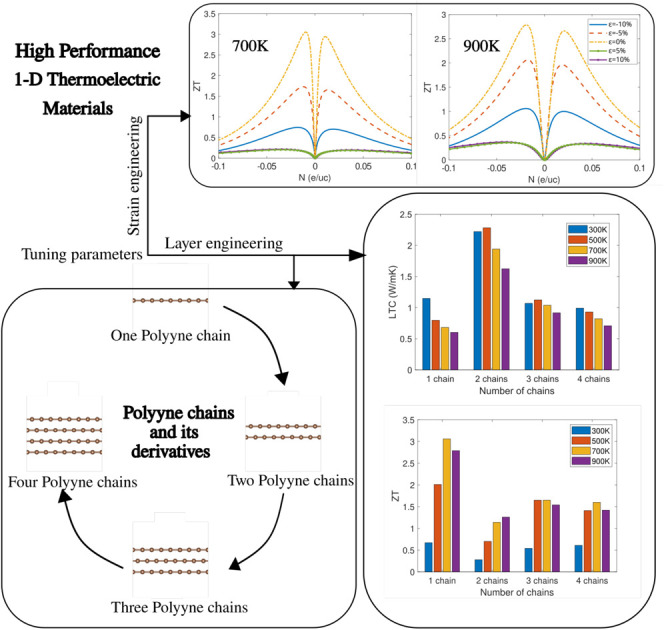

## I. Introduction

Thermoelectricity seeks to efficiently bridge the ubiquitous but low quality energy of heat with versatile but demanding electricity production, addressing the challenge of connecting these two forms of energy.^1^ Thermoelectric materials are environmentally friendly and can help mitigate environmental impacts following the Paris Climate Agreement because they boost energy sustainability by directly transforming waste heat into electricity.^2^ This is crucial because over two thirds of global energy utilization processes result in energy loss, primarily as waste heat.^3^ Thermoelectric generators do not use fossil fuels, emit harmful emissions, or have moving parts. They can be used for electronics cooling by removing excess heat from electronic components and converting it into electricity.^4^ Thermoelectric materials have been primarily constrained in their utilization for large-scale and niche applications due to their limited economic viability.^5^ The thermoelectric performance of a material is typically evaluated using the dimensional figure-of-merit (*ZT*),^6^ which is outlined below.
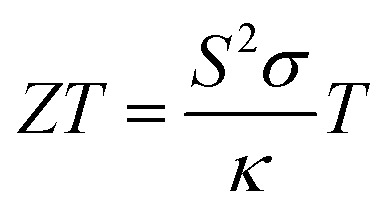


In this equation, *S*, *σ*, and *T* denote the Seebeck coefficient, electrical conductivity, and temperature in Kelvin, respectively. The total thermal conductivity (*κ*) is the sum of the electronic thermal conductivity (*κ*_e_) and the lattice thermal conductivity (*κ*_l_). The maximum *ZT* at a given temperature can be achieved by increasing the numerator (power factor – PF = *S*^2^*σ*) and decreasing the denominator, as indicated by the corresponding proportionality. The situation is more complicated than it appears because of conflicting correlations among the diverse physical quantities involved within the equation. This challenge arises because as the Seebeck coefficient decreases as a result of higher carrier concentrations, the electrical conductivity increases, subsequently leading to an increase in thermal conductivity.^7^ This adverse effect undermines efforts to enhance the *ZT* factor, which is crucial for thermoelectric performance. Therefore, the search for improved thermoelectric materials faces a major hurdle: finding systems with lower *κ* and higher *S* and *σ*.

Most of the industrially produced thermoelectric materials are bulk materials, namely Bi_2_ Ti_3_,^8^ PbTe,^9^ and Si–Ge alloys.^10^ They exhibit a *ZT* value of approximately 1 (ref. 11) at their optimal temperatures. Hicks and Dresselhaus^12^(1993) predicted that lower dimensionality benefits thermoelectric applications leveraging sharp density of states (DOS) peaks at the Fermi level, which was experimentally confirmed *via* quantum wells and wires.^13^ Furthermore, it influences lattice thermal conductivity by enhancing the scattering of phonons more effectively than electrons, primarily through increased boundary and interfacial scattering.^11^ As a result, novel two-dimensional (2D) materials like the graphenes,^14^ phosphorenes,^15^ penta-graphenes,^16^ MXenes,^17,18^ and transition metal dichalcogenides^19^ have been engineered and tailored to serve as future oriented high-performance thermoelectric materials. Transitioning into the quantum realm holds various compelling motivations. Numerous early innovative concepts, including the substantial improvement in power factor resulting from quantum confinement and density of states engineering, along with decreased thermal conductivity due to classical size effects, have been validated through experimental confirmation.^20^ The miniaturization of electronic devices poses challenges in finding suitable materials and managing increased levels of dissipated power. Increased phonon scattering at the surface and defects lowers lattice thermal conductivity, while electric conductivity remains unaffected, resulting in a notable enhancement in thermoelectric efficiency. Henceforth, various one-dimensional (1D) nanostructures,^21^ including nanowires (NW),^22^ nanotubes,^23^ and nanocomposites,^24^ have been reported to exhibit enhanced thermoelectric performance. The theoretical *ZT* value of Bi_2_Te_3_ NW with a diameter of 0.5 nm exceeds 14.^25^ With a diameter of just 0.8 nm, ZnO NW witnessed a staggering 30-fold surge in their *ZT* value compared to bulk.^26^ Experimental peaks in reported *ZT* values hover around 1 for individual Si NW,^27^ Bi_0.5_Sb_1.5_Te_3_ NW,^28^ Bi_2_(Te, Se)_3_ NW array thin films,^29^ and bulk nanostructured Bi_2_Te_3_ NW.^30^ However, for bulk nanostructured β-Zn_4_Sb_3_ NW,^31^ the reported *ZT* value reaches an impressive 1.59. Experimental measurements have also shown that the *ZT* values of 1D nanostructures utilizing Bi–Sb–Te–Se complexes fall within the range of 1.5 to 2.5.^32,33^

Our focus lies within the domain of carbon-based lower-dimensional materials. Carbon's versatile nature results in diverse allotropes which depends on its hybridization state and atomic arrangement,^34^ with carbyne featuring 1D sp-hybridized carbon atoms. The alternating single-triple bond polyyne structure is predicted to be favored by Peierls distortion over the cumulene structure, which consists solely of cumulated double bonds.^35^ The utilization of π-conjugated polymers in electronic devices, including but not limited to photovoltaics,^36^ transistor type memory devices,^37^ and various organic optoelectronic devices and sensors,^38^ finds its foundation in their unique electronic characteristics originating from the conjugation of π-orbitals along the molecular backbone. Several research groups^39,40^ have successfully isolated and characterized the linear polyynic framework, despite its synthetic challenges involving sp-hybridized carbon atoms, through persistent efforts. Recent theoretical work has outlined the significance of quantum anharmonicity in determining the phase transition from cumulene to polyyne.^41^ Polyynic C_18_ rings have been subjected to scientific inquiry to devise a range of logical gate functionalities.^42^ Moreover, investigations into the adsorption of Ag clusters onto C_18_ rings underscore the potential of these complexes in advancing the development of exceptionally efficient optoelectronic and non-linear optical devices.^43^ Buntov *et al.*^44^ indicated that thermal conductivity diminishes under strain and exhibits nearly linear augmentation with increasing chain length. Wang *et al.*^45^ suggested that for carbon atomic clusters linked by two aluminum electrodes, electric conductance was notably low within specific chemical potential ranges (−0.97 eV to −0.94 eV), resulting in the discovery of significant thermopower in this region. New advances in computational methods coupled with experimental verification can speed up the discovery of new thermoelectric material classes and impact of variations like defects, interfaces, dopants, and alloys—to further tailor transport behavior and maximize efficiency.^46^ A comprehensive study of the thermodynamic stability and thermoelectric properties of individual polyyne chains and their derivatives is necessary to predict their potential use as thermoelectric materials in low-dimensional devices.

In this study, we investigate the structural and electronic properties of one, two, three, and four polyyne chains, evaluating their potential utility in thermoelectric power generation. By employing electronic doping and strain effects at different temperatures, we aim to optimize the *ZT* of these materials. We analyze their Onsager coefficients, lattice thermal conductivity, and other key phonon properties such as heat capacity, phonon lifetime, and group velocity to understand their temperature dependence. Additionally, we examine their structural and thermodynamic stability through phonon dispersion analysis, formation energy calculations, and *ab initio* thermal stability simulations. Our comprehensive approach seeks to identify and characterize highly efficient and stable 1D thermoelectric materials for advanced energy harvesting technologies.

## II. Methodology

Vienna *Ab Initio* Simulation Package (VASP)^47,48^ was employed for computing pseudopotential plane-wave density-functional theory (DFT) calculations. To describe the electron exchange and correlation, the Perdew–Burke–Ernzerhof^49^ generalized gradient approximation (PBE-GGA) approach was chosen, and the ion cores were modeled using all-electron projector-augmented wave (PAW)^50,51^ pseudopotentials. The Brillouin zones were sampled *via* Γ-centered Monkhorst–Pack *k*-point meshes with 11 × 1 × 1 subdivisions. To account for van der Waals (vdW) interactions, the optB88-vdW functional^52^ was utilized. Tight constraints of 10^−4^ eV and 10^−3^ eV Å^−1^ on the electronic total energy and forces, respectively, were used to optimize all the structures accurately. To prevent any interactions between periodic images, sufficient vacuum (≥15 Å) was maintained in the two directions perpendicular to the chain. The Phonopy^53^ package was utilized to perform supercell density functional perturbation theory (DFPT)^54,55^ phonon calculations for the phonon dispersions. We have used the Nose–Hoover thermostat^56^ to maintain control of the temperature, *i.e.*, it keeps the system at a fixed number of particles (*N*), volume (*V*) and temperature (*T*) for the *ab initio* molecular dynamics (AIMD) calculations at 900 K for 2 ps. For the thermoelectric properties, BoltzTrap2 (ref. 57) package was used employing Boltzmann transport equations under constant scattering time approximation (CSTA) and rigid band assumption. Electronic properties and atomic structure plots were generated using sumo^58^ and black visualization for electronic and structural analysis (VESTA),^59^ respectively. Phono3py^60^ code was deployed to calculate the lattice thermal conductivities at different temperatures employing a 3 × 1 × 1 supercell with a *k*-mesh of 21 × 1 × 1. Phono3py auxiliary tools^61^ were used for cumulative lattice thermal conductivity, modal heat capacity, group velocity and phonon lifetime plots. Modal contributions to lattice thermal conductivity were analyzed graphically employing the phono3py-mode-plot code as a part of phono3py-power-tools^62^ package. Corresponding equations are as follows.

Cumulative thermal conductivity as a function of phonon frequency is defined as;
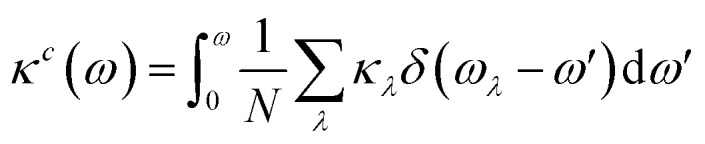
where *κ*_*λ*_ are the modal thermal conductivities, *ω* is the phonon frequency and *N* is the number of phonon wave vectors included in the summation.

Cumulative thermal conductivity as a function of mean free path (MFP) is defined as;
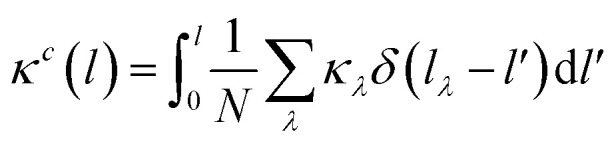
where MFP is defined in the single-mode relaxation time approximation (RTA) by a vector;**l**_*λ*_ = **v**_*λ*_*τ*_*λ*_.*l*_*λ*_ = |**l**_*λ*_| and modal thermal conductivity (*κ*_*λ*_), *i.e.* the contribution to *κ* from the phonon mode *λ* in the single-mode RTA is defined as
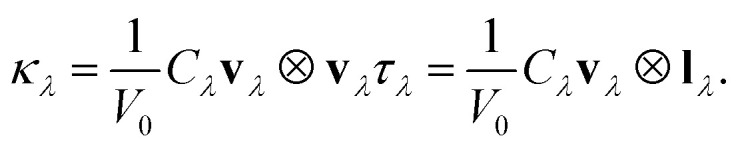


The macroscopic thermal conductivity tensor (*κ*_l_) is calculated^63^ as a sum of contributions from individual phonon modes *λ* according to:
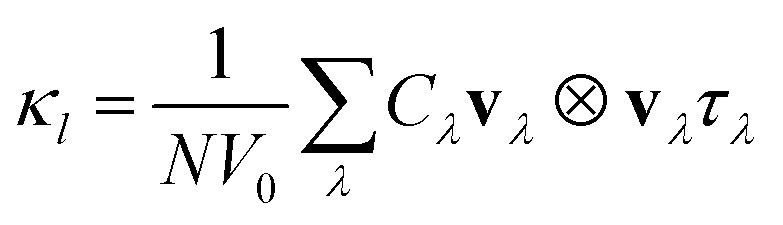
where *C*_*λ*_ are the (volumetric) heat capacities, **v**_*λ*_ ⊗ **v**_*λ*_ are the tensor products of the group velocities, *τ*_*λ*_ are the lifetimes, and *V*_0_ is the volume of the unit cell. *C*_*λ*_ and **v**_*λ*_ are calculated within the harmonic approximation and *τ*_*λ*_ are calculated as the inverse of the phonon linewidths *Γ*_*λ*_ as:
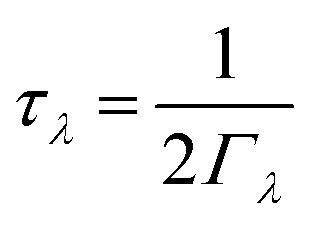


## III. Results and discussion

### A. Structural and electronic properties

We have used the most stable polyyne structure for our calculations, which features significant bond length alternation (BLA, *δ*_r_ = 0.046 Å) with alternating single and triple bonds. PBE-GGA functional, along with the structural parameters optimized by B3LYP in all computations, were used, as mentioned in Karthik *et al.*^64^ since B3LYP functional is recognized for providing the most accurate predictions for both the geometry and band gap of polyyne.^65^ Since obtaining accurate electronic properties is crucial for determining reliable thermoelectric properties, we conducted calculations employing both HSE and PBE functional to determine the band structure. We tried spin-polarized calculations for all the chained systems, but the system showed no effect on spin due to the completely filled p-orbitals. We calculate the electronic band structure along the high symmetry path Γ–X–Γ, in the first Brillouin zone. Due to the predicted closure in the band gap for AA-stacked chains,^66^ we modeled two, three, and four chains by vertically stacking one polyyne chain in AB, ABA, and ABAB configurations, respectively. Our work on polyyne derivatives was motivated by two main reasons. Firstly, in an experimental scenario, synthesis may result in multiple chains forming, and to our knowledge, there is no theoretical background for such derivative systems. Secondly, we hypothesized that incorporating more chains into the structure might increase the DOS effective mass, potentially enhancing thermoelectric efficiency.

**Fig. 1:**
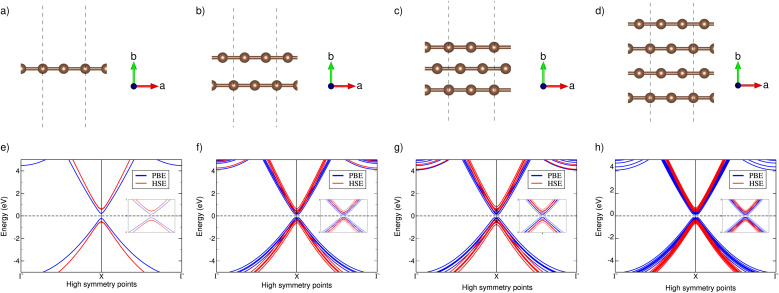
Optimised atomic structures of (a) one, (b) two, (c) three and (d) four polyyne chains and their respective band structures using PBE and HSE functionals (e–h).

Fig. 1 reveals that all the polyyne chain systems exhibit semiconducting behavior with direct PBE band gaps of 0.42, 0.30, 0.30, and 0.29 eV for 1, 2, 3 and 4 chains, respectively. Corresponding DOS plots are given in the ESI.† The PBE functional introduces an unphysical self-Coulomb repulsion, resulting in a systematic underestimation of band gaps.^67^ To achieve more accurate band gap predictions, we employed the Heyd–Scuseria–Ernzerhof functional (HSE06), which incorporates short-range exact Hartree–Fock exchange, significantly reducing the Coulomb self-repulsion error.^68^ They remained direct band gap semiconductors, with band gaps changing to 1.04, 0.54, 0.76, and 0.69 eV, respectively. Using two-band toy models for electronic structure, Chasmar *et al.*^69^ and Sofo *et al.*^70^ have demonstrated that the optimal thermoelectric materials should possess a band gap within the range of 6–10 *k*_B_*T* corresponding to 0.26–0.43 eV at 500 K and 0.36–0.6 eV at 700 K.

The band dispersions near the X point show parabolic behavior at low energies. The PDOS analysis (not shown here) indicates that the low-energy bands originate from carbon's p orbitals. In semiconductors, electrons experience less freedom of movement than in a vacuum. This reduced mobility is accounted for by assigning the electron an effective mass^71^ given by 
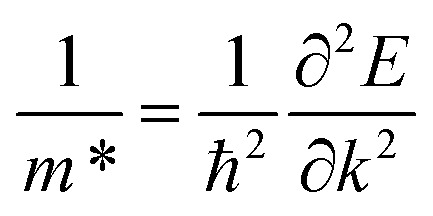
. In the band structure diagrams in Fig. 1, concave parabolas (VB) indicate holes, characterized by a negative effective mass, while convex parabolas (CB) indicate electrons, characterized by a positive effective mass. A steeper slope in these parabolas corresponds to a lower effective mass for the charge carriers due to lower group velocity. Therefore, it is clear from the figure that electrons (larger gradient) have a lower effective mass (0.02 m_0_) and, thus, higher mobility compared to holes (1.1 m_0_) which have a smaller gradient. Therefore, it would lead to enhanced electrical conductivity and increased electronic thermal conductivity, but the overall thermal conductivity will depend on the balance between electronic and lattice contributions, which can significantly affect the figure of merit.

In the case of a degenerate semiconductor, the Seebeck coefficient (*S*) is described by the expression 
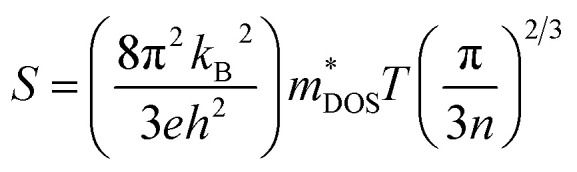
. Here, *k*_B_ denotes the Boltzmann constant, *e* represents the electronic charge, *h* is the Planck constant, and 
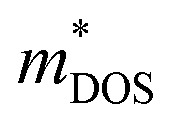
 is the effective mass of the density of states. A higher effective mass typically leads to a higher Seebeck coefficient because it increases the DOS at the Fermi level. Following the formula 
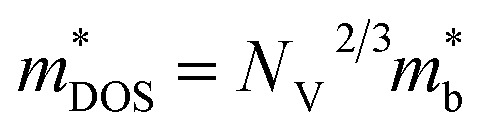
, where *N*_V_ encompasses orbital degeneracy and 
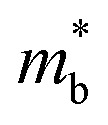
 represents the average DOS effective mass of the degenerate valleys, the presence of multiple degenerate valleys can yield a substantial DOS effective mass without significantly compromising the mobility *μ*.^72^ Analysis of band structure diagrams in Fig. 1 suggests that with an increase in the number of chains, there is a corresponding rise in the number of bands near the Fermi level. As illustrated in the inset of Fig. 1, higher polyyne derivative systems exhibit degenerate states near the Fermi level. The abundance of states around the Fermi level in valence and conduction regions suggests their potential for thermoelectric applications as they increase the valley degeneracy and band effective mass, improving the Seebeck coefficient.

### B. Structural and thermodynamic stability analysis

From a practical application standpoint, stability is crucial to ensure the integrity of the chains. The methodologies employed here to ensure the structural stability of the polyyne chains include formation energy calculation, phonon spectra calculation, and AIMD simulation. Since we previously assessed the thermodynamic stability of one and two polyyne chains in our prior work,^64^ we have extended the analysis to include systems with three and four chains.

Formation energy provides valuable insights into the stability and synthesis of compounds and materials, aiding researchers in understanding and predicting their behavior. The formation energy of polyyne chain derivative systems were calculated using the equations:*E*_f_ = *E*_tot_*n*__ − *nE*_iso_where *n* denotes the number of chains, *E*_tot_*n*__ represents the total ground state energy of the *n*-chain structure, and *E*_iso_ denotes the ground state energy of the isolated chain.

The results presented in Table 1 indicate that the formation energy remained negative as the number of chains increased. As the chain count increases from two to four and the inter-chain distance increases, the formation energies significantly drop, becoming more negative. This trend suggests that the structure remains stable and becomes more energetically favorable with an increasing number of chains. Reducing the inter-chain distance enhances vdW interactions, which in turn facilitates charge transfer and improves electron transfer across the interface,^73^ which could potentially enhance the material's thermoelectric capabilities.

**Table 1:** Number of chains (*n*), corresponding formation energy (*E*_f_) values and inter-chain distance (*d*)

*n*	*E*_f_ (eV)	*d* (Å)
Two	−0.042	3.64
Three	−0.086	3.91, 3.67
Four	−0.132	3.90, 3.69

**Fig. 2:**
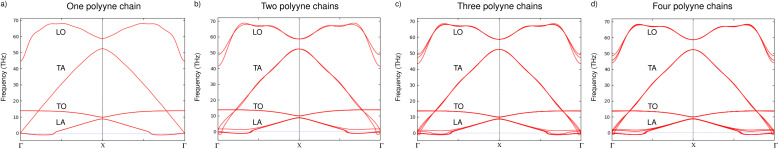
Phonon dispersion relations for (a) one (b) two (c) three and (d) four polyyne chains using DFPT calculations.

Ensuring the dynamic stability of systems is crucial for their experimental utilization across various applications. Phonons represent quantized vibrational modes within a crystal lattice, and their dispersion relation delineates the propagation of these vibrational modes in the momentum space. Negative frequencies indicate the presence of imaginary components in these vibrational modes, suggesting that the lattice vibrates with exponentially growing amplitudes. This leads to instability and the eventual collapse of the crystal structure. Fig. 2 illustrates the phonon spectra of various systems of polyyne chains. There are negative frequencies around the Γ point that are extremely small, well below 1 THz, which is the accuracy limit. These imaginary branches observed in the DFPT results stem from computational artifacts and do not signify dynamical instabilities.^74,75^ Phonon band splitting is clearly observable for higher phonon frequencies for all multi-chain systems, particularly for the longitudinal optical (LO) mode, while other modes remain largely unaffected by inter-chain interaction.

**Fig. 3:**
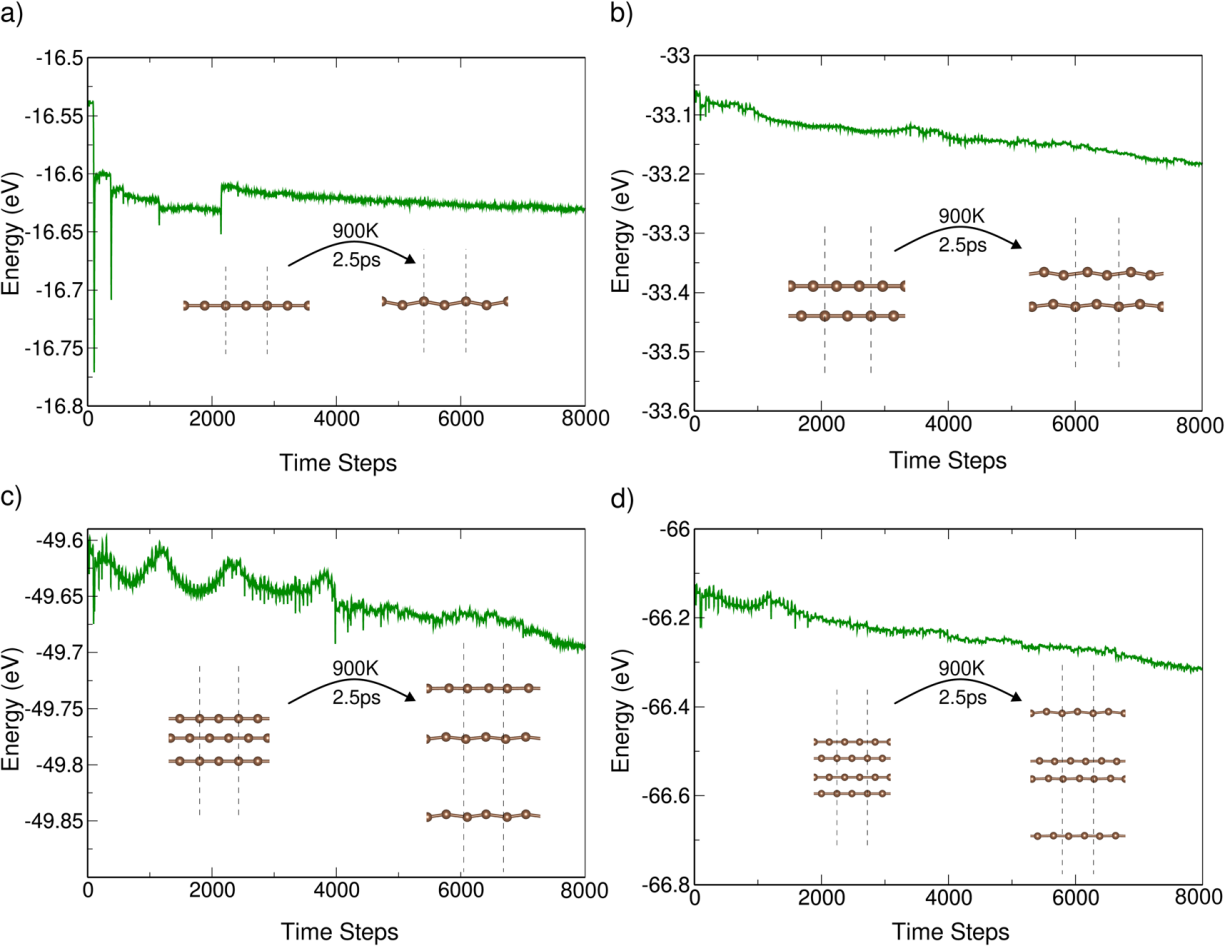
Energy *vs.* time steps for (a) one (b) two (c) three, and (d) four polyyne chains using AIMD simulations.

Given that the systems are exposed to temperatures as high as 900 K, we have performed AIMD simulations for all the systems at this temperature. During the initial time steps, there were significant fluctuations in the ground state energy. Still, the energy has stabilized by the end of 2.5 picoseconds (ps), as shown in Fig. 3. The structures (as shown in the inset) also exhibit minimal structural distortions at the end of the simulation.

### C. Thermoelectric properties

**Fig. 4:**
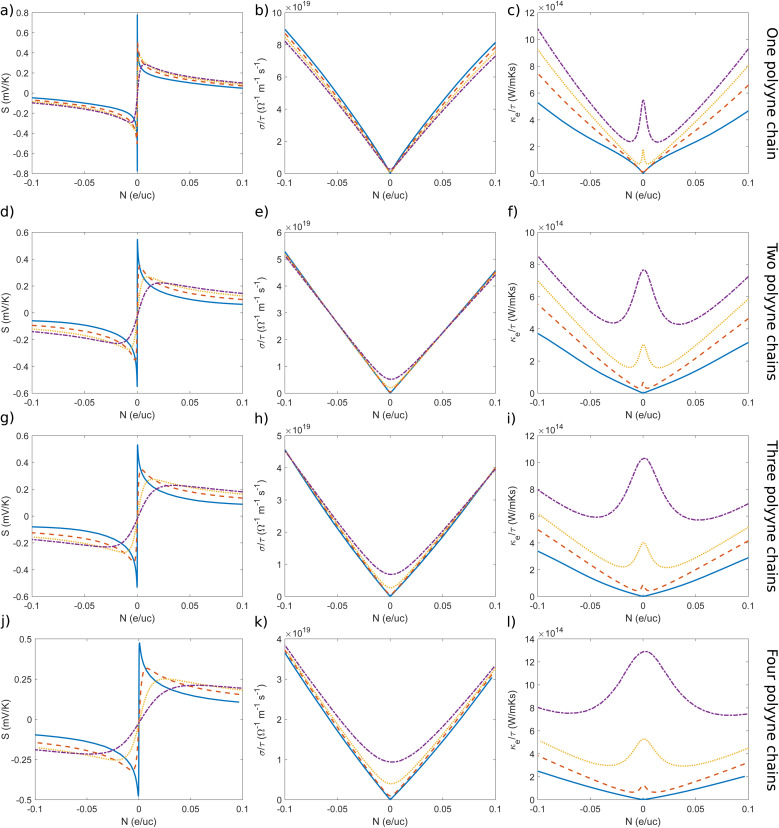
Seebeck coefficient (*S*), electrical conductivity (*σ*/*τ*), and electronic thermal conductivity (*κ*_e_/*τ*) respectively for one (a–c), two (d–f), three (g–i) and four (j–l) polyyne chains calculated using PBE functional.

The thermoelectric efficiency of any material can be linked through its figure of merit, *ZT*. Utilizing the computed band structures, we have determined the electronic transport coefficients of all systems, including the Seebeck coefficient (*S*), electrical conductivity (*σ*/*τ*), electronic thermal conductivity (*κ*_e_/*τ*) and *ZT* (total figure of merit). For comparison purposes, we opted for a relaxation time (*τ*) value of 10^−14^ s across all doping levels, as the *ZT* maxima occur near zero doping levels. At the same time, the lattice thermal conductivities at various temperatures, including relaxation time, were computed using Phono3py. We have investigated the influence of doping on these coefficients by varying the charge carrier concentration (*N*). Negative and positive *N* indicate electron/n-type and hole/p-type doping, respectively. The thermoelectric coefficients of different chain configurations across different temperature and doping ranges are presented in Fig. 4.

Analyzing the Onsager coefficients for one polyyne chain, it can be observed that the electronic thermal conductivity (*κ*_e_/*τ*) increases with temperature. However, the lattice thermal conductivity (*k*_l_) decreases with increasing temperature, as plotted in Fig. 7, along with the Seebeck coefficient. At higher doping concentrations, electrical conductivity (*σ*/*τ*) decreases with increasing temperature, whereas at near zero doping concentrations, electrical conductivity increases with temperature. Therefore, overall, the *ZT* increases for higher temperature ranges. For temperatures above 500 K, the *ZT* values are significantly bigger than 1 for one polyyne chain and its derivatives, which is comparable to standard thermoelectric materials.^76^ The peak value of 3.06 is achieved at 700 K with an electron carrier concentration of −0.010 e per uc, while for hole doping, the value decreases to 2.94 at a carrier concentration of 0.011 e per uc. This peak in *ZT* further reinforces the previously mentioned optimal band gap range of 0.36 to 0.6 eV at 700 K for achieving maximum thermoelectric performance. The additional peaks at various temperatures and their respective charge carrier concentrations are tabulated in Table 2. We attempted to maximize the *ZT* across a broad doping range; however, the peak *ZT* was observed within a narrower doping regime. Notably, while *ZT* peaks are observed in both doping regimes, they are slightly higher in the electron-doping regime. Previously, it was anticipated that the overall *ZT* would increase in electron doping regimes due to the lower effective mass and higher mobility of electrons. This outcome is advantageous as it aligns well with typical experimental doping conditions, facilitating direct comparisons.

**Table 2:** Maximum *ZT* and corresponding charge carrier concentrations for one, two, three, and four polyyne chains at different temperatures

Temperature (K)	*N* (e per uc)	PBE *ZT*_max_	*N* (e per uc)	HSE *ZT*_max_
**One polyyne chain**				
300	−0.014	0.67	−0.012	0.81
500	−0.007	2.01	−0.008	2.24
700	−0.010	3.06	−0.008	3.66
900	−0.019	2.79	−0.006	4.99

**Two polyyne chains–AB stack**				
300	−0.014	0.28	−0.015	0.26
500	−0.018	0.70	−0.014	0.64
700	−0.026	1.14	−0.015	1.23
900	−0.046	1.26	−0.017	1.82

**Three polyyne chains–ABA stack**				
300	0.015	0.54	−0.011	0.44
500	0.021	1.25	−0.012	0.95
700	0.041	1.65	0.014	1.71
900	0.073	1.54	0.015	2.65

**Four polyyne chains–ABAB stack**				
300	−0.017	0.61	0.015	0.53
500	0.031	1.41	0.018	1.46
700	0.067	1.60	0.016	2.69
900	−0.100	1.42	0.022	3.81

The band gap in the AA stacked chains is minimal, prompting us to transition to the AB-type stacks (AB, ABA, and ABAB), aligning with our goal of enhancing thermoelectric efficiency. In the AB stacked two chains scenario, there is a noticeable decline in the Seebeck coefficient compared to one chain system. For electrical conductivity, higher doping concentrations lead to a minuscule decline with increasing temperature, while near zero doping concentrations result in a small increase. In contrast, the values of electronic thermal conductivity shows same trend as before with slightly lower values at higher doping concentrations compared to one polyyne chain. These trends directly correspond to a reduction in *ZT* values. Similar to the one-chain system, the maximum *ZT* of 1.26 is observed in the electron-doped regions (*N* = −0.046 e per uc). In the hole-doped regions (*N* = 0.052 e per uc), the maximum *ZT* is slightly lower, at 1.22. Therefore, opting for electron doping rather than hole doping would be advantageous in such systems to enhance thermoelectric efficiency. Both charge carrier concentrations are higher than those corresponding to the *ZT* peaks of one polyyne chain. The other maxima at different temperatures and their respective charge carrier concentrations are listed in Table 2. The total *ZT* values for several additional stacking configurations, achieved by sliding one of the chains from the AA stacking by a sliding parameter *s* are provided in the ESI,† because of all, the AB stacking is the most energetically stable arrangement.

Transitioning from three to four chains, despite the increase in electrical conductivity near zero doping levels, there is a corresponding rise in electronic thermal conductivity, which doesn't lead to a significant increase in *ZT*. There is only a slight increase in *ZT* for all temperatures. In the three-chain system, electrical conductivity shows less variation with temperature at higher doping levels, whereas in the four-chain system, electrical conductivity increases with temperature. It's worth noting that the doping charge carrier concentration at *ZT* maxima is higher when transitioning to three and four polyyne chains. Thus, there must be a trade-off between doping concentrations and maximizing *ZT* because optimizing doping levels is crucial to achieving the best thermoelectric performance. We believe that making internal adjustments in the stacking patterns of such systems would be a good approach to maximize *ZT*.

**Fig. 5:**
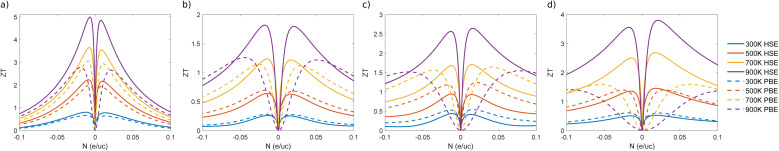
Figure of merits for (a) one, (b) two, (c) three, and (d) four polyyne chains calculated using HSE and PBE functionals.

Since the PBE functional underestimates the band gap, we performed band structure calculations using the HSE functional and used it to calculate all thermoelectric coefficients and the figure of merit. Since the band gap predicted by HSE is higher than that obtained from PBE, the Seebeck coefficient increases significantly, while electrical conductivity exhibits a slight increase. However, corresponding changes in electronic thermal conductivity counteract this effect. Therefore, the primary factor contributing to the increase in *ZT* is likely the enhancement of the Seebeck coefficient. The corresponding *ZT* values obtained from both functionals are shown in Fig. 5, while other plots of thermoelectric coefficients are provided in the ESI.† In the case of a single polyyne chain, the highest *ZT* (4.99 at 900 K) is achieved at a low carrier concentration of *N* = −0.006 e per uc. However, as the number of chains increases from one to four, the peak *ZT* value reaches 3.81 at 900 K, but this occurs at a higher carrier concentration of *N* = 0.022 e per uc.

#### 1. Strain effect on one polyyne chain

**Fig. 6:**
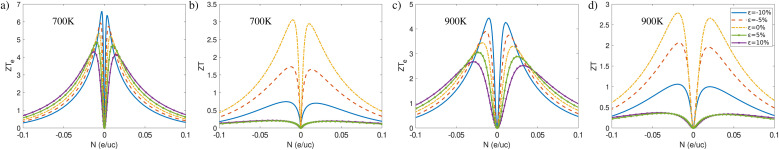
Electronic figure of merit (*ZT*_e_) and total figure of merit (*ZT*) at 700 K (a and b) and 900 K (c and d) respectively for one polyyne chain under different strained conditions calculated using PBE functional.

Given that fabrication processes can induce mechanical strain and impact the electronic properties of the system, we investigated the effect of various strain regimes on both electronic and total figure of merit values, as illustrated in Fig. 6. Uniaxial strain was applied along the chain direction, with the remaining two directions maintained in vacuum. In this configuration, *l*_0_ denotes the initial lattice parameter, and *l* refers to the strained lattice parameter, expressed by the formula 
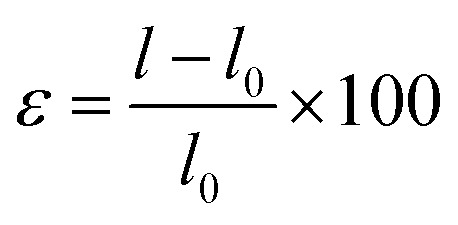
. Higher temperatures of 700 K and 900 K were selected because the *ZT* maxima were observed at these temperatures.

At 700 K, the polyyne chain under compressive strain exhibits promising *ZT* values (*ZT* = 1.7 at *ε* = −5%), highlighting its potential for thermoelectric devices. Conversely, the *ZT* value drops significantly under tensile strain (*ZT* = 0.2 at *ε* = 5%), suggesting that tensile strain should be avoided during device fabrication. The corresponding peak *ZT* at 900 K is 2.1 (*ε* = −5%) and 0.4 (*ε* = 5%), respectively. Although both types of strains decrease the total figure of merit, tensile strain shows a more significant decline due to substantial increases in lattice thermal conductivity. Given the similarities in electronic properties, we anticipate observing comparable effects in strained cases across other multi-chained systems.

### D. Lattice thermal conductivity and related phonon properties

**Fig. 7:**
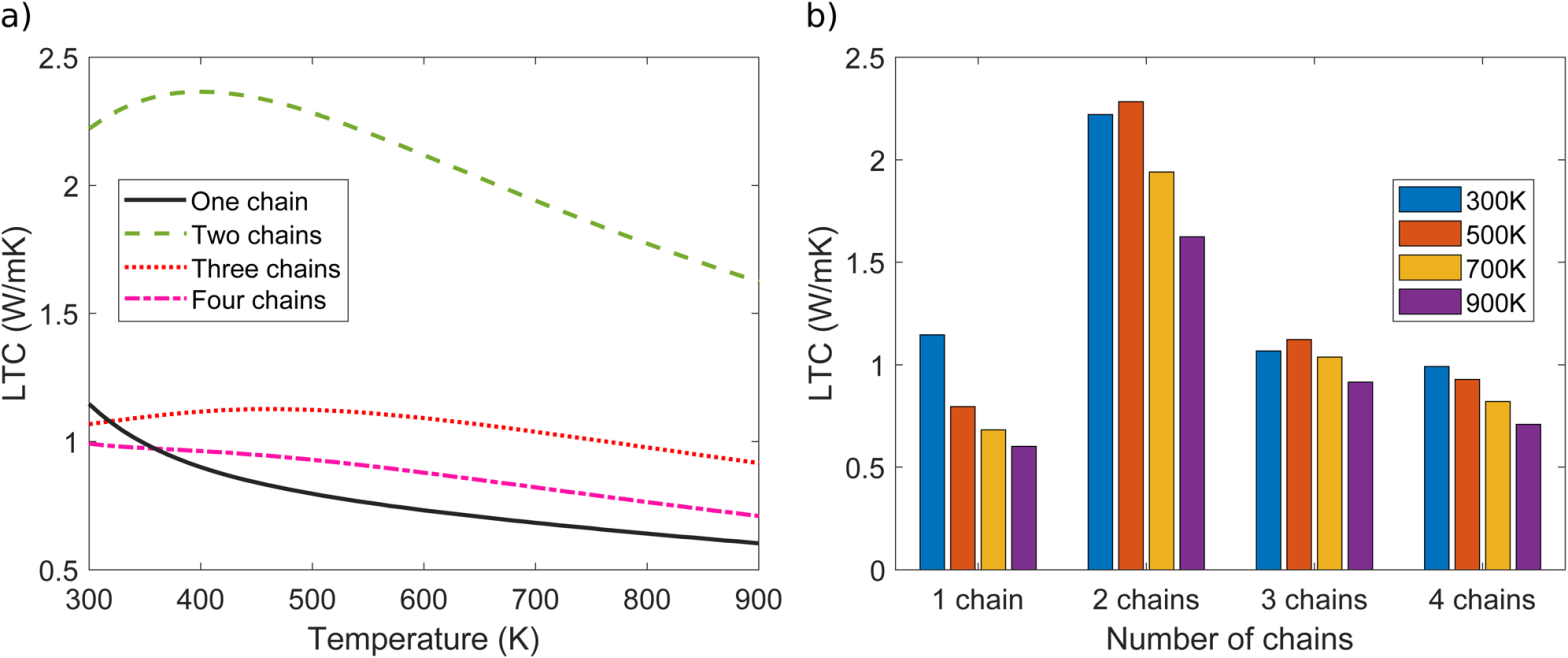
(a) LTC as a function of temperature and (b) LTC as a function of number of chains for one, two, three, and four polyyne chain systems.

The relationship of lattice thermal conductivity (LTC) for a 1D material from the kinetic theory^77^ is expressed as:
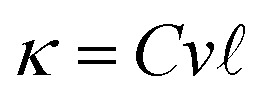
where *C* denotes the heat capacity per unit volume, *v* represents the average phonon velocity, and 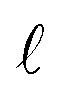 signifies the mean free path (MFP) of phonons. LTC was computed for one, two, three and four polyyne chains under comparable conditions for analysis as in Fig. 7. There is a trend of decreasing LTC with increasing temperature from 300–900 K, primarily due to enhanced phonon–phonon scattering, which reduces phonons' MFP. The results show that LTC decreases with increase in number of chains from two to four. The room temperature (300 K) LTC values are 1.15, 2.22, 1.07, and 0.99 W mK^−1^ for one, two, three, and four chains, respectively. From Fig. 7b, it is evident that the difference in LTC between the three and four-chain systems is relatively minor compared to the variations observed in other systems.

**Fig. 8:**
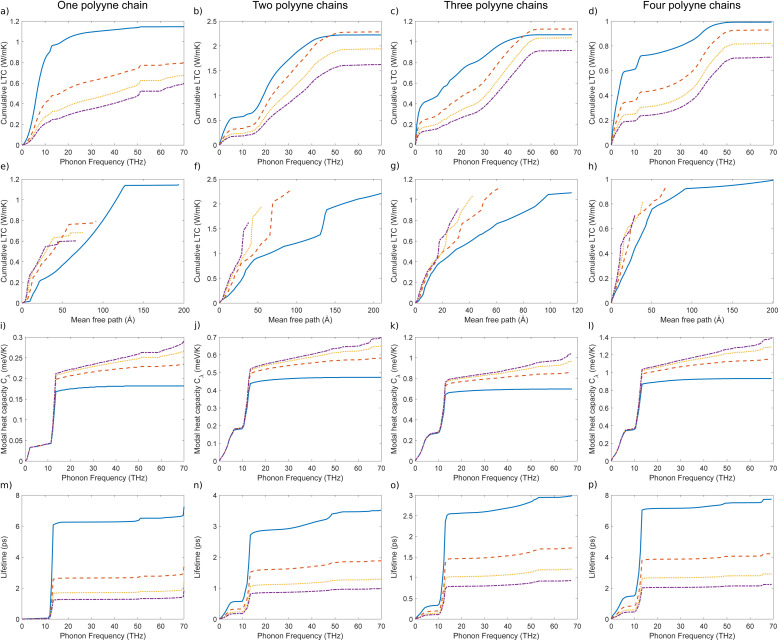
Cumulative LTC as a function of phonon frequency (a–d) and mean free path (e–h) and modal heat capacity (i–l) and lifetime (m–p) as a function of phonon frequency of one, two, three and four polyyne chain systems calculated using phono3py-kaccum.

To investigate how the contribution to the total LTC of material accumulates as a function of phonon frequency, we plotted cumulative lattice thermal conductivity against phonon frequency for different temperatures, as illustrated in Fig. 8a–d. This visualization offers insights into how phonons with varying characteristics contribute to the overall LTC. For all systems and at all temperatures, more than half of the peak LTC is achieved before 30 THz, indicating a significant contribution from acoustic modes. In the case of one polyyne chain, the first peak appears around 14 THz after a steep increase in LTC, with minor variations due to temperature changes. This marks transitioning from longitudinal acoustic (LA) to transverse optical (TO) modes. From 14 to 51 THz, the LTC increases gradually, showing a lesser contribution from the transverse acoustic (TA) mode, with another peak at 52 THz. This peak corresponds to the end of the TA mode, and the region from 52 to 59 THz shows no increase in LTC, indicating the gap (at X point) between the TA and longitudinal optical (LO) modes in the phonon dispersion relation. Beyond this, the LTC increases further due to the contribution of the LO mode, eventually saturating around 70 THz. Another important feature can be observed in multi-chain systems: a smaller, nearly zero gradient between 5 and 10 THz, which indicates the end of LA modes. For three- and four-chain systems, LTC saturates at lower frequencies, which demonstrates that the contribution of high-frequency LO and TA modes to LTC is minimal.

To investigate the effect of the mean free path (MFP) of phonons on LTC, we plotted the cumulative LTC against MFP at various temperatures, as illustrated in Fig. 8e–h. The initial gradient of the graph is steep, while towards the end, it converges. This implies that phonons with shorter MFP (acoustic) lead to a more rapid increase in LTC, while phonons with longer MFPs (optical) typically contribute less to the rate of LTC increase. The longer MFP of optical phonons is due to their lower scattering rates, which stem from their higher energy and lower density of states at lower frequencies. At lower temperatures, phonons exhibit relatively long MFP due to the reduced scattering events. As the temperature ascends, the number of phonons increases, leading to more frequent Umklapp scattering events, thereby shortening the MFP. For one polyyne chain, the results show that 50% of the LTC is contributed by phonons with a MFP shorter than 76 Å at 300 K, which decreases to 12 Å at 900 K. In multi-chained systems, these values shift from 79–24 Å, 32–17 Å, and 33–10 Å for two, three, and four polyyne chains, respectively, across the 300–900 K temperature range. This indicates that phonons with short MFPs (acoustic phonons) contribute more significantly to LTC than those with longer MFPs. There is a tiny increase in MFP as the number of chains increases from one to four, *i.e.* from 194 Å to 201 Å at 300 K but it decreases from 66 Å to 29 Å at 900 K. As the number of chains increases, more vibrational modes with longer wavelengths emerge due to band splitting. These longer-wavelength modes are less prone to phonon–phonon scattering, leading to an increase in MFP at lower temperatures. For one polyyne chain, the peak LTC reaches MFP up to 194 Å at 300 K but decreases to 66 Å at 900 K. Similar trends are observed in multi-chained systems, with MFP values ranging from 212 to 38 Å, 116 to 32 Å, and 201 to 29 Å for systems with two, three, and four polyyne chains, respectively.

To investigate the influence of phonon frequency on the heat capacity of a material, we plotted modal heat capacity against phonon frequency at various temperatures, as illustrated in Fig. 8i–l. For all polyyne chain systems at all temperatures, the steepest increase in the gradient occurs between 10 and 15 THz, where the TO mode becomes dominant. Lower frequency LA and TO modes contribute significantly to the heat capacity, more so than the higher frequency TA and LO modes. It is evident that temperature does not play a significant role in the values of modal heat capacity in the lower frequency regime; however, beyond this point, as the temperature increases, the modal heat capacity also increases. As the temperature rises from 300 K to 900 K, there is a corresponding increase in peak modal heat capacity, which ranges from 0.18 to 0.3 meV K^−1^ for one polyyne chain, 0.47 to 0.7 meV K^−1^ for two chains, 0.7 to 1.0 meV K^−1^ for three chains, and 0.93 to 1.4 meV K^−1^ for four polyyne chains. This increase can be attributed to the greater number of vibrational modes resulting from additional branches especially in the LO mode in the phonon dispersion curve where degeneracies are lifted. These multiple modes enhance the material's ability to store energy, thereby increasing the heat capacity. This would have elevated the LTC with an increased temperature since heat capacity is directly proportional to it. However, the rate at which the heat capacity (*C*_*λ*_) increases might be significantly lower compared to the reduction in the MFP. Consequently, the overall effect is a decrease in LTC with increasing temperature.

To gain deeper insights into the underlying mechanism of decrease in MFP, it is beneficial to examine the lifetimes of phonon modes as functions of phonon frequency, as illustrated in Fig. 8m–p. This was crucial because the MFP is directly proportional to the phonon lifetime. Lower-frequency phonons (LA and TO) exhibit shorter lifetimes owing to the significant scattering, but as the phonon frequency increases, the lifetime values increase and tend to saturate. As the temperature rises, phonon lifetimes decrease due to increased scattering. For temperatures ranging from 300 K to 900 K, the cumulative lifetime peak values for one polyyne chain range from 7.9 to 2.4 ps. In comparison, for two, three, and four polyyne chains, these values shift from 3.5 to 1.0 ps, 3.0 to 0.94 ps, and 7.8 to 2.2 ps, respectively. It is noteworthy that during the periods of *C*_*λ*_ spikes (between 10 and 15 THz), there are corresponding inverted spikes in the phonon lifetime plots according to temperature. This, in turn, reduces the MFP more and LTC with an increase in temperature as *C*_*λ*_ increase is comparatively less. For all systems and at all temperatures, the lifetime values saturate after 15 THz, marking the end of the TO mode. This indicates that the high-frequency TA and LO modes do not contribute to the increase in phonon lifetime.

**Fig. 9:**
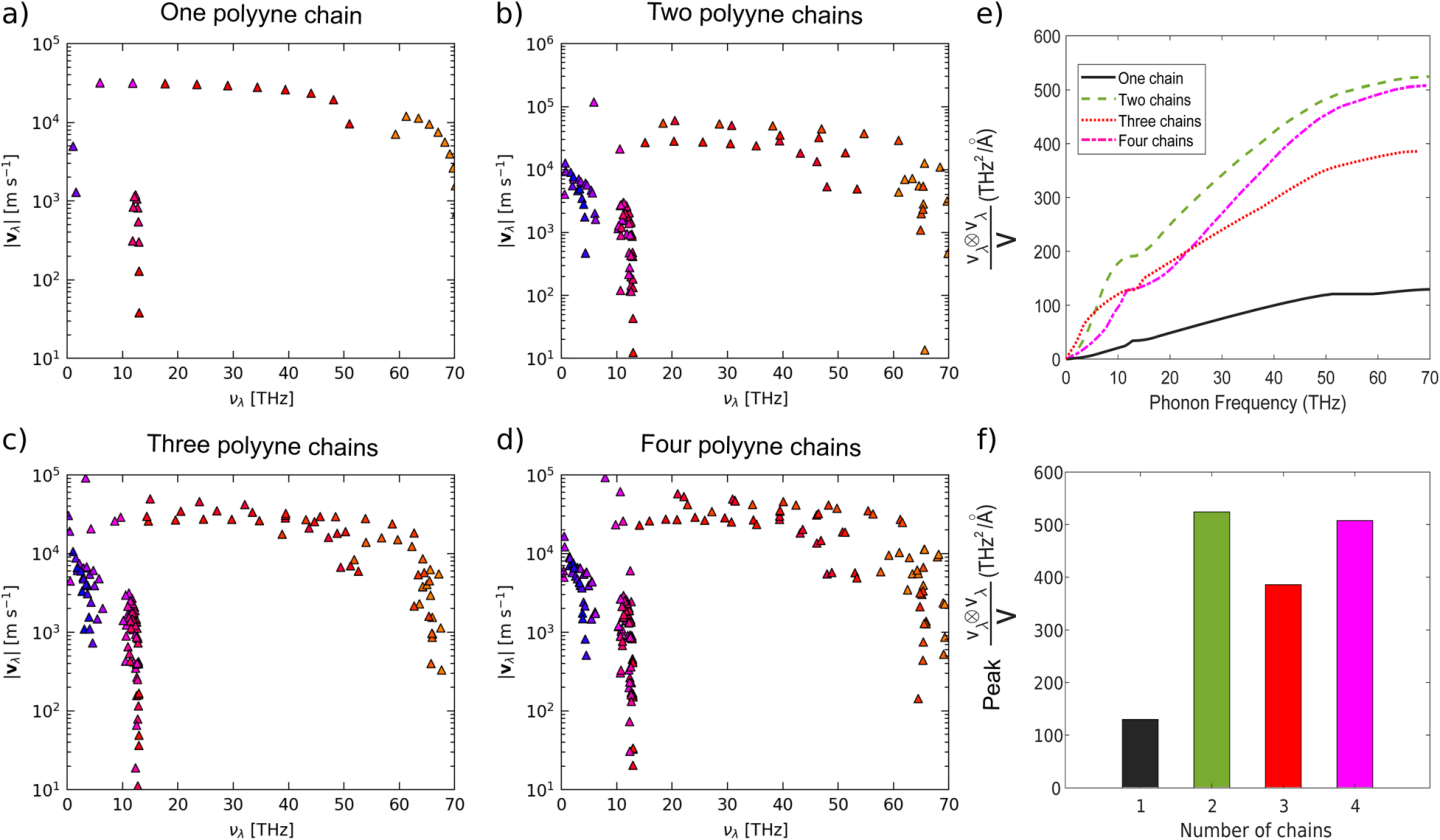
Frequency spectrum of group velocity norms (|*v*_*λ*_|) of (a) one (b) two (c) three, and (d) four polyyne chain systems and outer product of group velocity divided by primitive cell volume as a function of (e) phonon frequency and (f) number of chains using phono3py-kaccum and phono3py-mode-plot, respectively.

Given the pivotal role of group velocity in shaping LTC, we plotted the frequency spectrum of group velocity norms, as in Fig. 9a–d. Phonons with higher group velocities possess a heightened ability to propagate energy efficiently throughout the crystal lattice as they can traverse longer MFP, augmenting LTC. In these systems, most acoustic phonons exhibit lower group velocities, with values ranging from 10^2^ m s^−1^ to 10^3^ m s^−1^ for one and two chains and from 10^3^ m s^−1^ to 10^4^ m s^−1^ for three and four chains. In contrast, the corresponding group velocity values for optical phonons fall within the 10^4^ m s^−1^ to 10^5^ m s^−1^ range across all systems. These observations align with the less steep LA phonon bands and the steeper LO phonon bands observed in Fig. 2. Given that previous sections have established that LTC is primarily contributed by acoustic phonons, their lower group velocities are a key factor contributing to the lower LTC value. Additionally, we have also plotted the frequency spectrum of group velocity weighted by the averaged thermal conductivity (Fig. 6 in the ESI†). This approach provides insights into the contributions of modes with varying group velocities to the overall thermal conductivity for different numbers of chains. From that figure it can be inferred that, although the overall group velocity increases in the case of four polyyne chains, its contribution to the LTC remains negligible. As a result, the LTC of the four-chain system is lower than that of the three-chain configuration. For all the systems, temperature has no impact on the norms of the group velocity.

LTC can be expressed as a function of heat capacity, phonon group velocity, and phonon lifetime as*κ* = *Cv*^2^*τ*

**Fig. 10:**
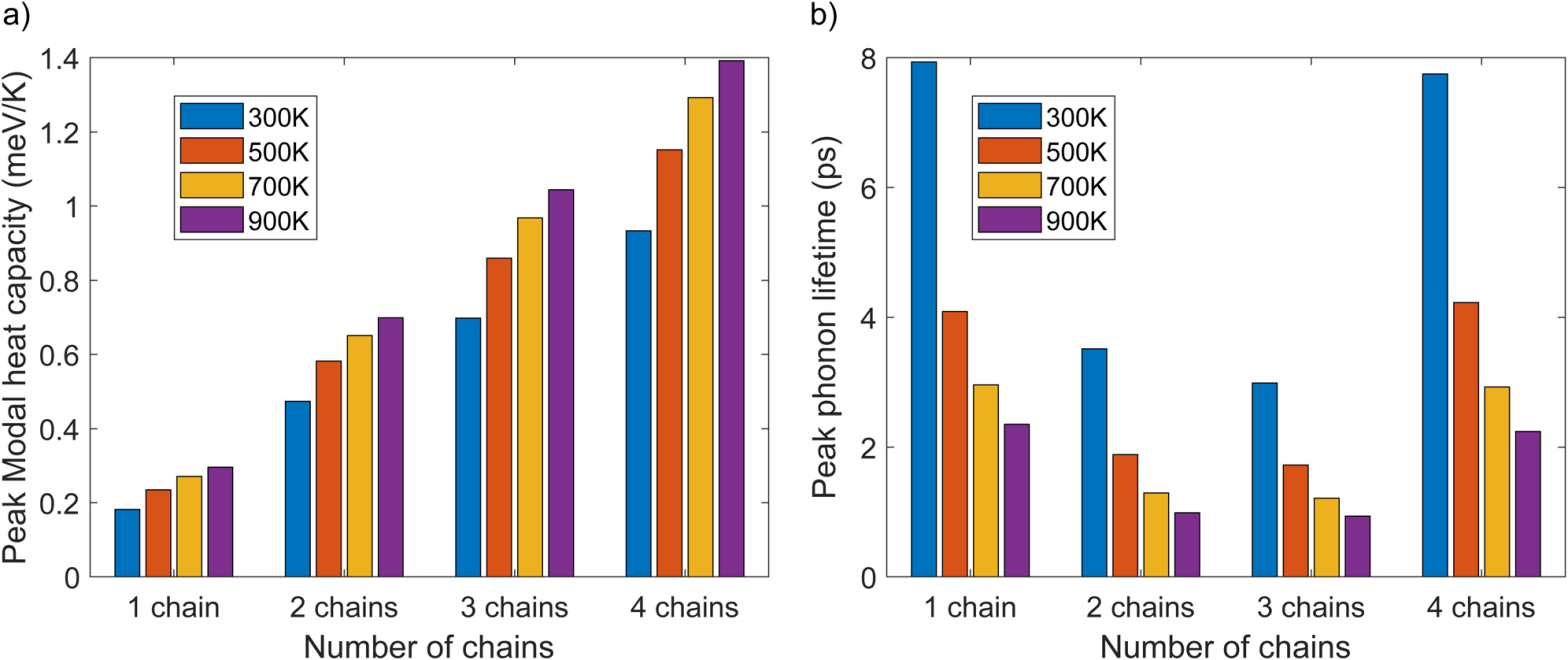
(a) Peak modal heat capacity and (b) peak phonon lifetime as a function of number of chains for one, two, three, and four polyyne chain systems for different temperatures using phono3py-kaccum.

From Fig. 10, it can be observed that the peak modal heat capacity increases significantly when transitioning from one chain to four chains, while the phonon lifetime has a different trend. Additionally, Fig. 9f indicates a substantial increase in group velocity from one to two chains. This rise in group velocity can be linked to the higher LTC in the two-chain system compared to the one-chain system. For the three-chain system, there is a decrease in phonon lifetime, accompanied by an increase in modal heat capacity and decrease in group velocity. As a result, the phonon lifetime and group velocity becomes the primary factors driving the decrease in LTC in this case.

**Fig. 11:**
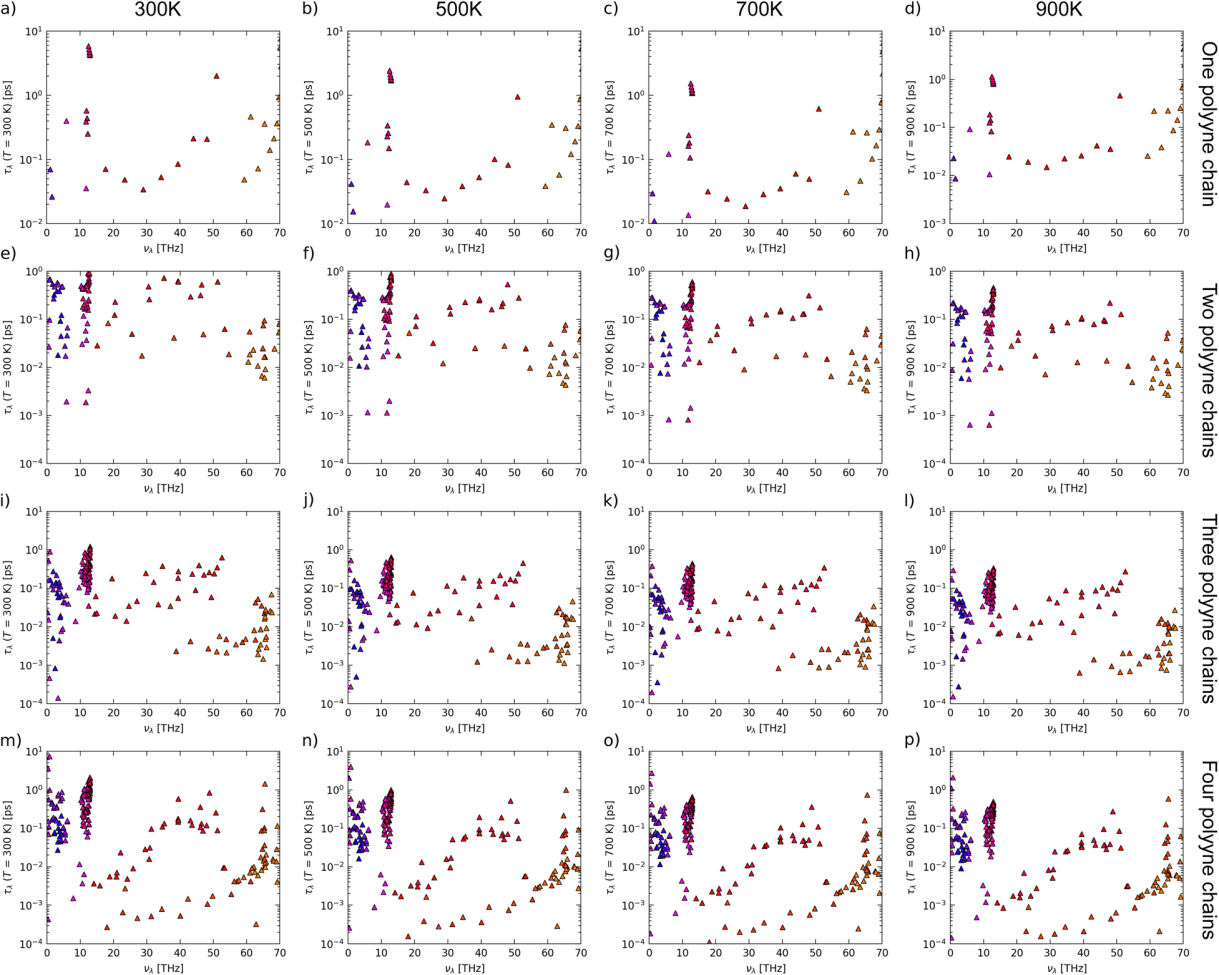
Frequency spectrum of phonon lifetimes of one [(a)–(d)], two [(e)–(h)], three [(i)–(l)] and four [(m)–(p)] polyyne chain systems at different temperatures using phono3py-mode-plot.

To examine the impact of phonon lifetimes on LTC, we plotted phonon lifetimes against frequency across different temperatures as depicted in Fig. 11. Phonons with longer lifetimes scatter less frequently, enhancing the material's ability to conduct heat and thereby increasing the LTC. It is indicative from the figure that, for one polyyne chain, the maximum lifetime of the acoustic branches reaches up to 6 ps at 300 K but drops to as low as 1 ps at 900 K, resulting in a reduction in the LTC. A similar trend is observed in multi-chained systems, with lifetimes ranging from approximately 3.5 to 0.9 ps, 5 to 1.7 ps, and 2 to 0.6 ps for systems with two, three, and four polyyne chains, respectively, as the temperature increases from 300 K to 900 K. As temperature rises, the lifetimes of all phonon modes decrease due to increased Umklapp scattering, driven by a higher phonon population and anharmonic interactions. This results in a decrease in thermal conductivity with increasing temperature. We observe that the relaxation time of acoustic branches peaks when the frequencies are in the range of 0–14 THz. This suggests that low-frequency LA phonons could play a significant role in contributing to the total LTC. When comparing the phonon lifetime densities across all systems, the highest density is also observed for those LA modes, with their density increasing at higher temperatures. This reinforces the fact that LTC is predominantly contributed by LA phonons. As seen from Fig. 11, the density of optical phonons also increases with temperature similar to LA phonons, but their contribution to LTC is minimal as they have comparatively lesser lifetimes, even at lower temperatures, and can be safely disregarded.

**Fig. 12:**
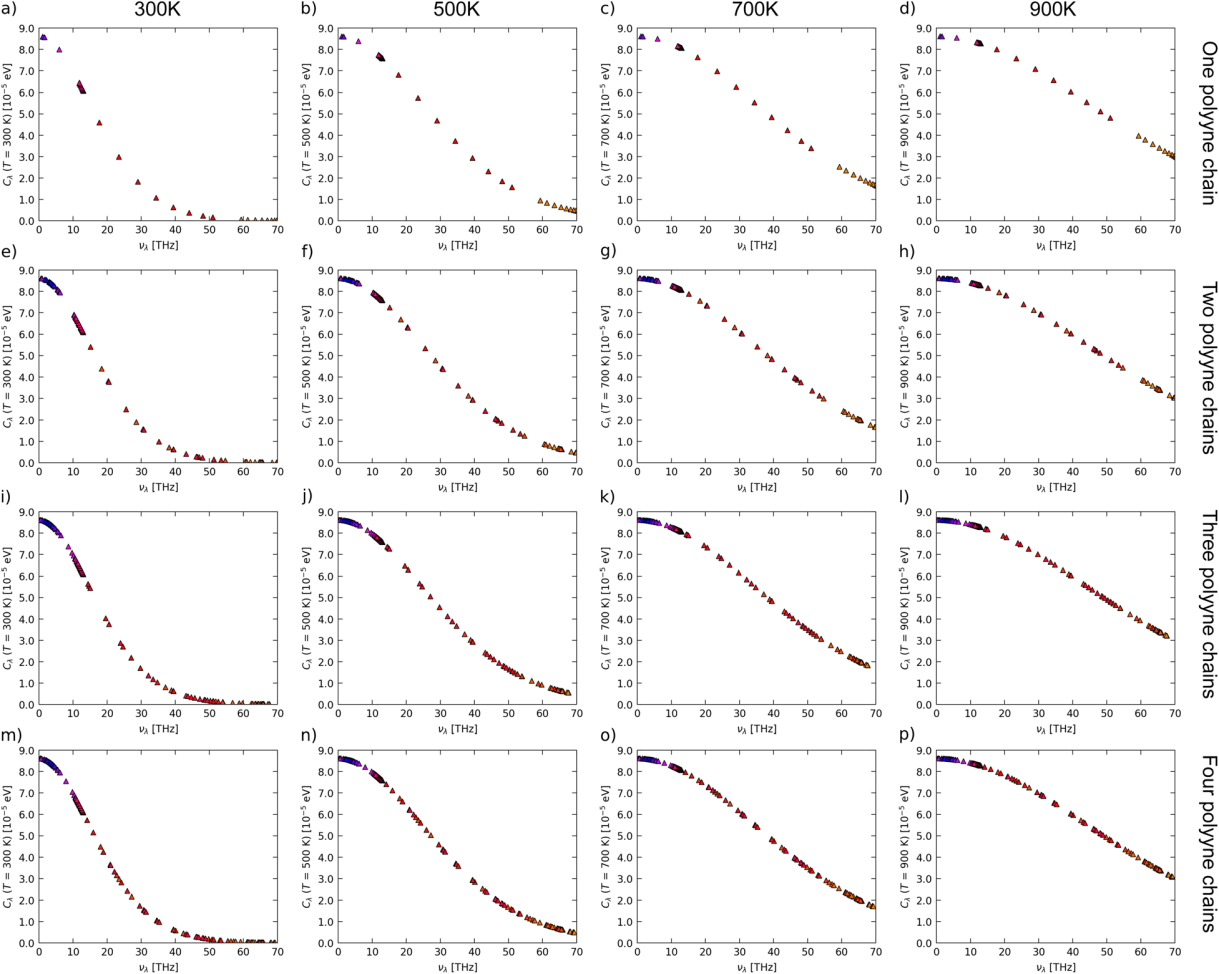
Frequency spectrum of heat capacity of one [(a)–(d)], two [(e)–(h)], three [(i)–(l)] and four [(m)–(p)] polyyne chain systems at different temperatures using phono3py-mode-plot.

The heat capacity (*C*) is temperature-dependent in many materials, subsequently impacting the LTC. As temperature increases, variations in the heat capacity may occur due to changes in the lattice structure or vibrational modes. These variations can alter the efficiency of phonon transport, thereby influencing the LTC. We have offered a visualization of the frequency spectrum of the heat capacity as in Fig. 12. As temperature increases, heat capacity exhibits a rising trend, as explained earlier. This increase predominantly influences high-frequency (optical) modes, while its effect on lower-frequency (acoustic) modes is minimal. Since high-frequency modes contribute less significantly to LTC, the overall impact of this increase is obscured by substantial reductions in phonon lifetimes and mean-free paths. In addition, we have weighted individual modes based on their average thermal conductivity in ESI† to further highlight the contribution of different modes to overall thermal conductivity.

In this study, we found that all polyyne chains and their derivative systems exhibit strong thermoelectric properties, even under strain and with an increasing number of chains. Notably, a single polyyne chain achieves impressive *ZT* values above 500 K, while all derivative systems show significant performance at temperatures above 700 K. The key factor influencing the change in LTC was determined to be the acoustic modes. These systems hold potential for applications in energy-efficient nanoelectronics, where thermoelectric energy harvesting plays a vital role.

## IV. Conclusion

In conclusion, we have demonstrated an ultra-high figure of merit (*ZT*) in a class of pure carbon based 1D materials – polyyne chain and its derivatives. Our meticulous studies on doping and temperature effects revealed that n-type doping is the most effective in maximizing *ZT* values for one and two chains while p-type doping proves promising for three and four chains in the temperature range of 500–900 K. Given the significant role of lattice thermal conductivity, we examined determining factors such as phonon lifetimes, group velocity, and mean free path, which further highlighted the crucial impact of acoustic modes. Impressively, all systems exhibited promising *ZT* values exceeding 1, underscoring their potential for thermoelectric power generation. We assessed their thermodynamic and structural stability through phonon dispersion studies, *ab initio* molecular dynamics simulations, and formation energy analyses as stability is paramount for their integration in various applications and devices. Considering potential strain during fabrication, our analysis showed that *ZT* values remain above 1 even under different strained conditions, further supporting their practical utility. When growing polyyne chains experimentally, it is common to observe multiple chains. Our study demonstrates that, even as the number of chains increases, the thermoelectric efficiency remains suitable for high-performance thermoelectric applications. Additionally, the low-dimensional nature of our systems facilitates seamless integration into miniature circuitry, enhancing their efficiency in converting dissipated heat into electricity.

## Data availability

All relevant data are within the manuscript and its additional files. The structures explored in this work have been deposited in the public repository https://github.com/Karthik-PhD/Polyyne_Files. The cif files of these structures, that were simulated using DFT, are provided in this repository. Further clarification on data is available upon reasonable request from the authors.

## Author contributions

Karthik H. J. (first author): conceptualization, data curation, methodology, investigation, formal analysis, writing – original draft, review & editing. Swastibrata Bhattacharyya (corresponding author): conceptualization, methodology, validation, formal analysis, resources, supervision, writing – review & editing.

## Conflicts of interest

The authors declare that they have no known competing financial interests or personal relationships that could have appeared to influence the work reported in this paper.

## Supplementary Material

NA-OLF-D4NA00998C-s001
