# Supplementary material for: High-performance one-dimensional thermoelectric materials: polyyne chains and their derivatives
Source: Nanoscale Adv. 2025 May 22;7(14):4381–96. doi: 10.1039/d4na00998c (PMC12146783; doi:10.1039/d4na00998c)
Supplement: NA-007-D4NA00998C-s001 [file NA-007-D4NA00998C-s001.pdf]

Supplementary Information (SI) for Nanoscale Advances.
This journal is © The Royal Society of Chemistry 2025

High-Performance 1D Thermoelectric Materials: Polyyne Chains and Their Derivatives
(Supplementary Information)

Karthik HJ and Swastibrata Bhattacharyya*

Department of Physics, Birla Institute of Technology and Science Pilani, Zuarinagar, Goa 403726, India

(Dated: April 20, 2025)

I. ELECTRONIC PROPERTIES - BAND STRUCTURE AND DENSITY OF STATES

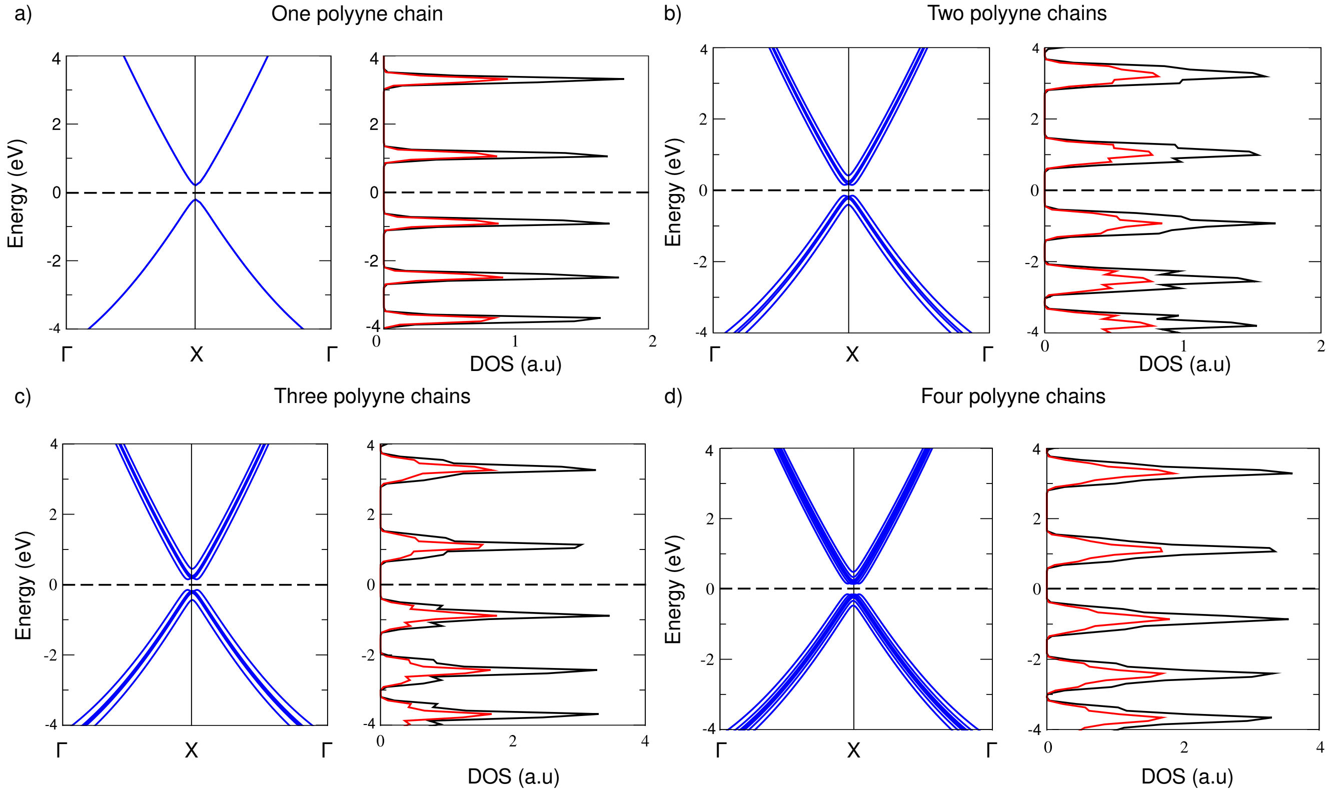

FIG. 1: Spin independent band structure and density of states for a) One polyyne chain, b) Two polyyne chains, c) Three polyyne chains and d) Four polyyne chains

*Corresponding author. Tel: +91-832-2580-365. E-mail: swastibratab@goa.bits-pilani.ac.in

II. CHAINS FEATURING DISTINCT STACKING CONFIGURATIONS ACHIEVED VIA SLIDING

We constructed a few additional stacking configurations by sliding a chain from the AA stacking configuration by a certain amount in the chain's direction; the sliding parameter (s) equates to the slide number times the lattice parameter. Fig 2 illustrates that the largest lattice thermal conductivity exists for slide 0.4, or s = $1.02\mathring{A}$. ZT is minimum for that sliding distance in accordance. The trend of ZT with sliding shows an initial increase, reaching a maximum at slide 0.3. Afterward, it dips before increasing again at slide 0.5. The corresponding opposite trend is observed in LTC values, suggesting that in such systems, LTC has a significant effect on determining the thermoelectric performance. Despite the highest ZT (1.73) being achieved at slide 0.5 at 900K with an electron carrier concentration of 0.039, there is a close value of 1.70 at slide 0.3, albeit at a lower temperature and with a smaller electron carrier concentration. Fig 3 illustrates the phonon lifetimes at different temperatures for all sliding cases. The changes in phonon lifetimes are in accordance to the changes in LTC.

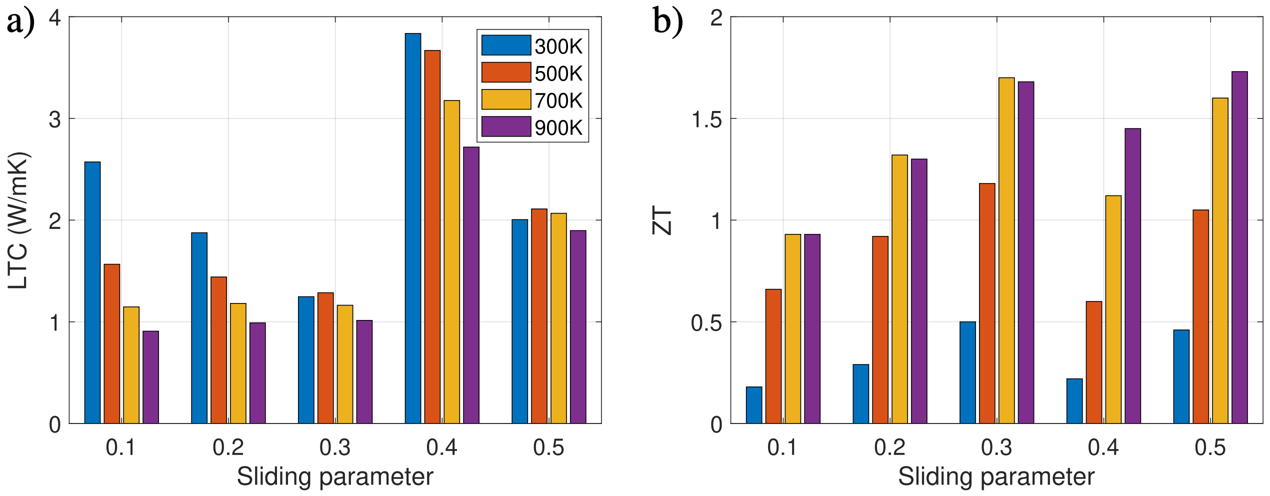

FIG. 2: Plots of sliding parameter versus a) ZT b) LTC

System	Temperature (K)	N (e/uc)	Max ZT
Slide 0.1	300	-0.012	0.18
	500	-0.014	0.66
	700	-0.027	0.93
	**900**	**-0.043**	**0.93**
Slide 0.2	300	-0.013	0.29
	500	-0.013	0.92
	**700**	**-0.020**	**1.32**
	900	-0.040	1.30
Slide 0.3	300	-0.011	0.50
	500	-0.012	1.18
	**700**	**-0.020**	**1.70**
	900	-0.033	1.68

System	Temperature (K)	N (e/uc)	Max ZT
Slide 0.4	300	-0.020	0.22
	500	-0.024	0.60
	700	-0.024	1.12
	**900**	**-0.034**	**1.45**
Slide 0.5	300	-0.017	0.46
	500	-0.018	1.05
	700	-0.023	1.60
	**900**	**-0.039**	**1.73**

TABLE I: Maximum values of ZT and corresponding charge carrier concentrations for different sliding distances at different temperatures

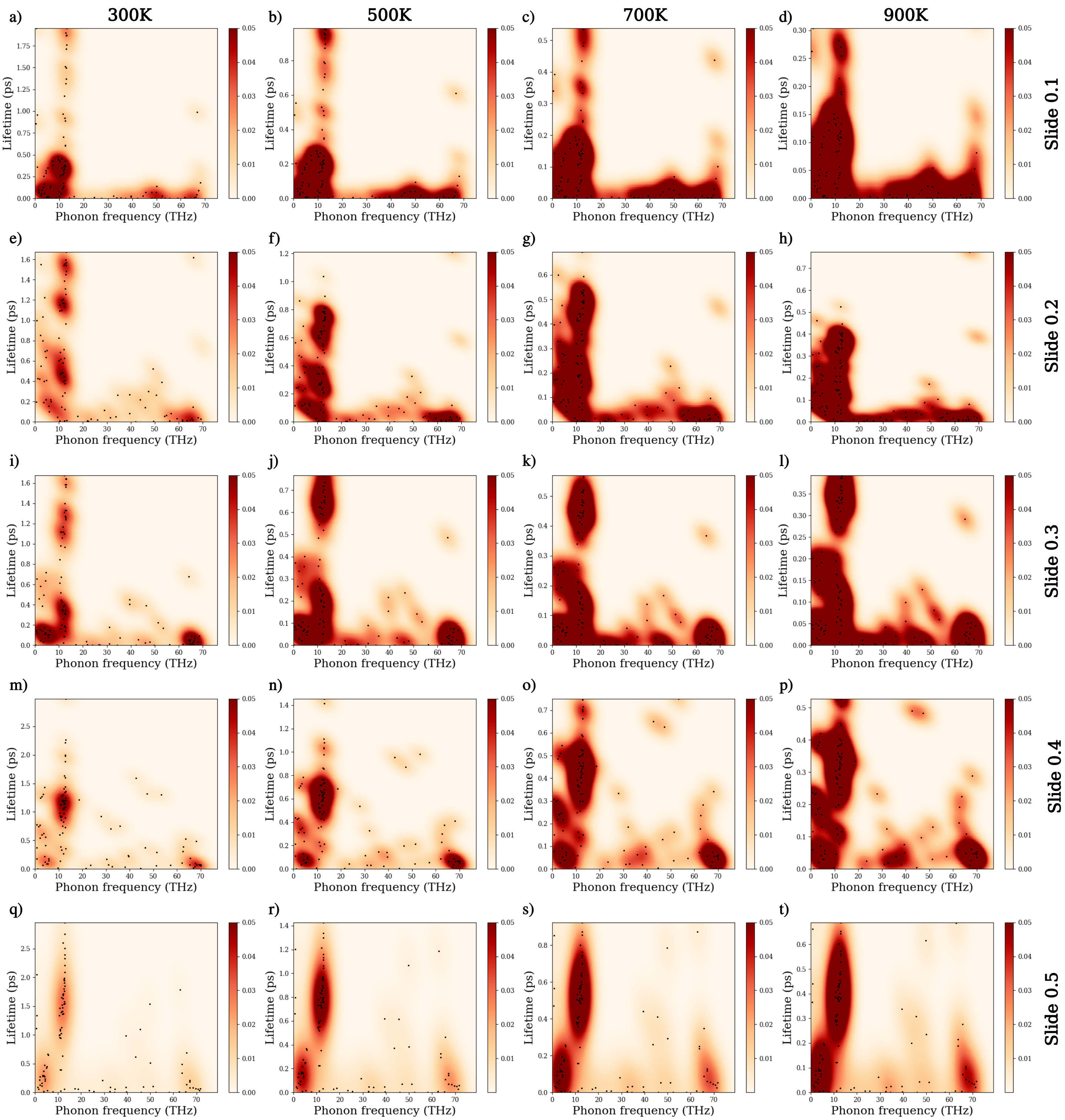

FIG. 3: Frequency spectrum of phonon lifetimes of all sliding cases of two chains

III. THERMOELECTRIC COEFFICIENTS

Fig 4 presents the thermoelectric coefficients of one, two, three and four polyyne chain systems calculated from the HSE band structures.

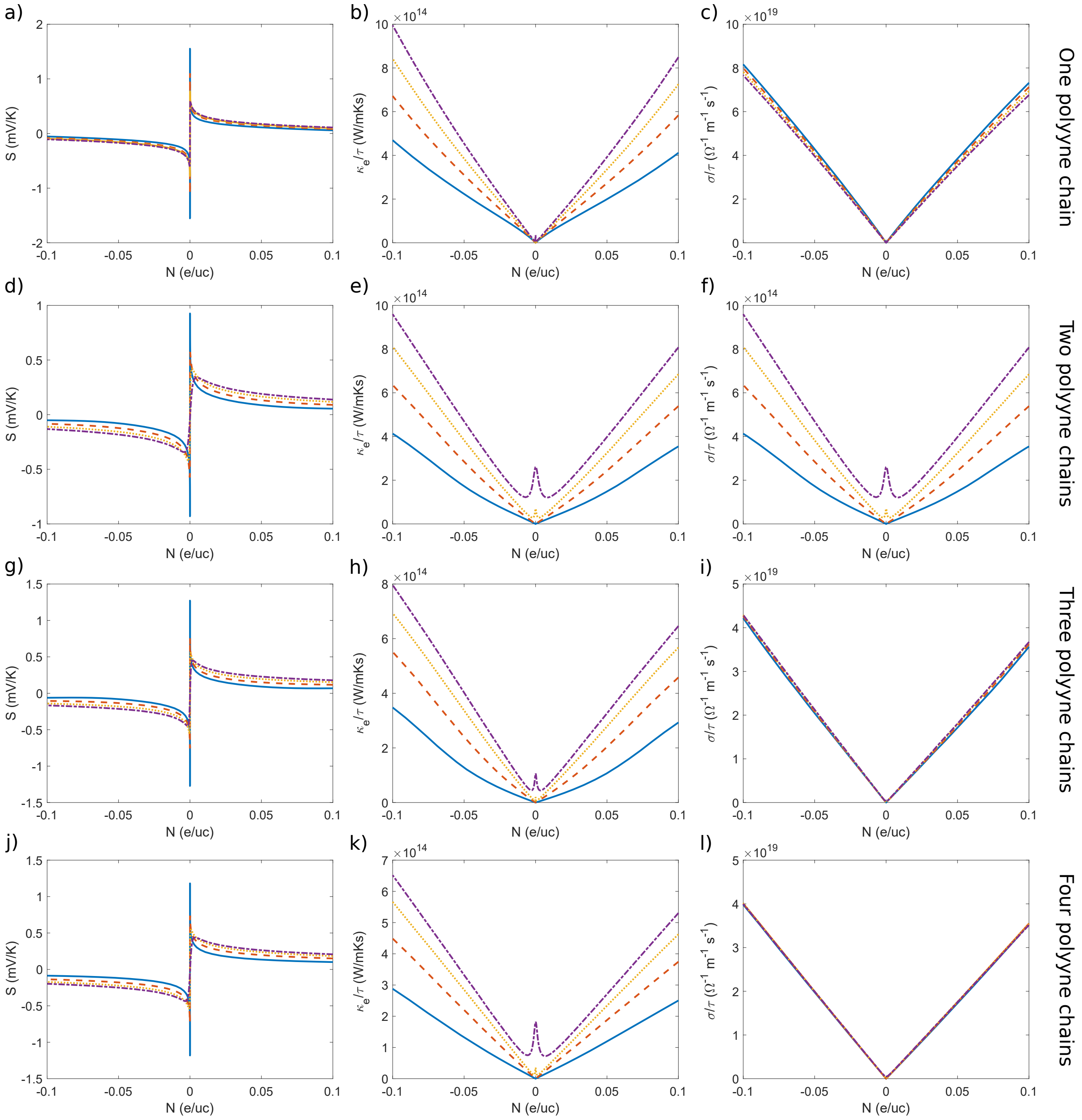

FIG. 4: Seebeck coefficient (S), Electronic thermal conductivity (κ_e/τ) and Electrical conductivity (σ/τ), respectively for one [(a-c)], two [(d-f)], three [(g-i)] and four [(j-l)] polyyne chains using HSE functional

Thermoelectric coefficients and total ZT for a modelled bulk system of polyyne chains using PBE functional is illustrated as in Fig 5

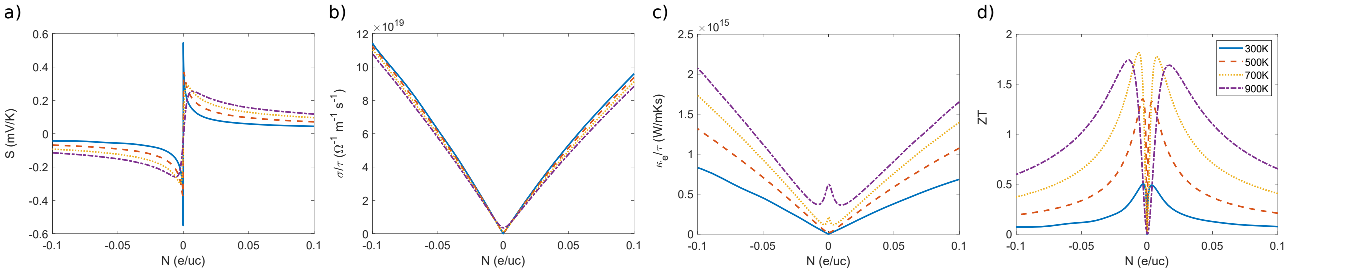

FIG. 5: a) Seebeck coefficient (S), b) Electrical conductivity (σ/τ), c) Electronic thermal conductivity (κ_e/τ) and d) Figure of merit (ZT) respectively for bulk system using PBE functional

IV. CONTRIBUTION OF DIFFERENT MODES

Contribution of different phonon modes to LTC due to changes in group velocity and heat capacity are illustrated in Fig 6 and Fig 7 respectively.

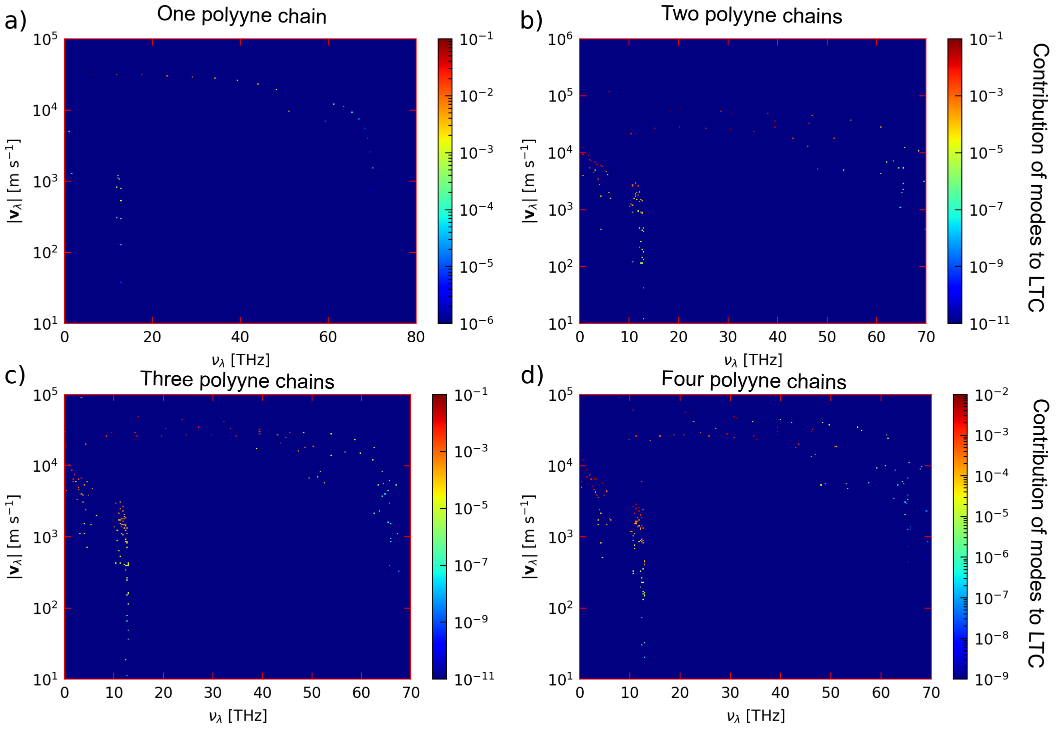

FIG. 6: Frequency spectrum of group velocity (v_λ) weighted by the averaged thermal conductivity of a) one b) two c) three, and d) four polyyne chain systems

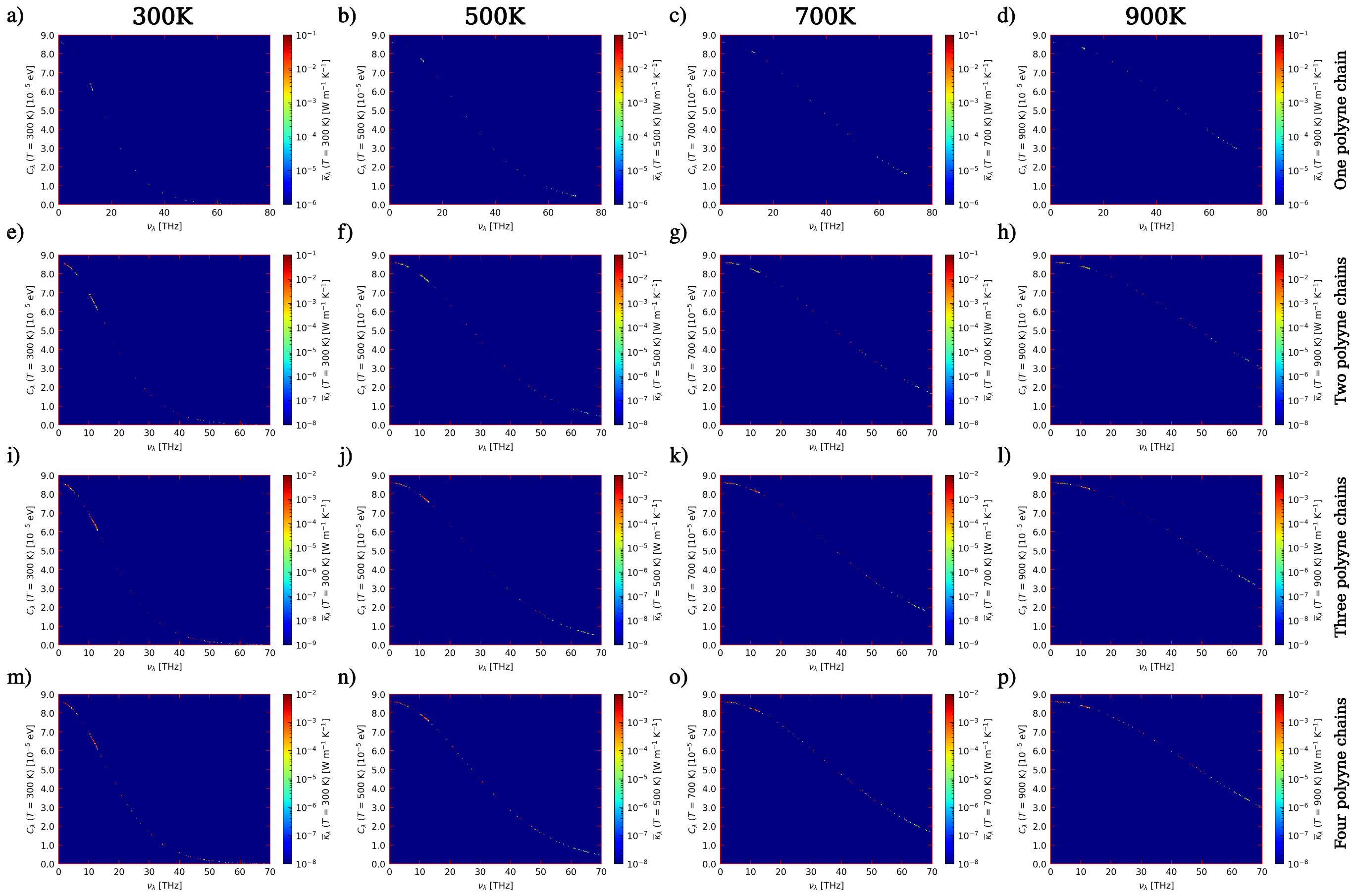

FIG. 7: Frequency spectrum of heat capacity weighted by the averaged thermal conductivity of a) one b) two c) three, and d) four polyyne chain systems
